# *Staphylococcus succinus* Infective Endocarditis, France

**DOI:** 10.3201/eid3003.230986

**Published:** 2024-03

**Authors:** Louise Ruffier d’Epenoux, Erwan Fayoux, Frédéric Laurent, Pascale Bémer, Raphaël Lecomte, Thierry Le Tourneau, Aurélie Guillouzouic, Stéphane Corvec

**Affiliations:** Centre Hospitalier Universitaire, Nantes, France (L. Ruffier d’Epenoux, E. Fayoux, P. Bémer, R. Lecomte, T. Le Tourneau, A. Guillouzouic, S. Corvec);; Université de Nantes, INSERM INCIT UMR 1302, Nantes (L. Ruffier d’Epenoux, S. Corvec);; Université de Nantes, CNRS, INSERM UMR 1087, l’Institut du Thorax, Nantes (T. Le Tourneau);; Université Claude-Bernard Lyon 1, Lyon, France (F. Laurent);; Groupement Hospitalier Nord, Lyon (F. Laurent);; Hôpital Croix Rousse, Lyon (F. Laurent)

**Keywords:** *Staphylococcus succinus*, infective endocarditis, antimicrobial resistance, *mec* gene, penicillin-binding proteins, France

## Abstract

Infective endocarditis is a rare condition in humans and is associated with high illness and death rates. We describe a case of infective endocarditis caused by *Staphylococcus succinus* bacteria in France. We used several techniques for susceptibility testing for this case to determine the oxacillin profile.

*Staphylococcus succinus* was first described in 1998 and was isolated from 25- to 35-million-year-old Dominican amber (*1*). Members of this species are widespread in nature. Studies have reported the frequent isolation of *S. succinus* bacteria from various sources, such as cheeses, dry or fermented meat products, the Dead Sea, and occasionally human specimens (*2*–*4*). We report a case of *S. succinus* infective endocarditis in a patient in France who had many cardiovascular risk factors: age, sex, hypertension, dyslipidemia, diabetes, and weight. In accordance with legislations in France and Europe, the use of anonymous data does not need approval of an ethics committee. 

On hospital day 1, an 83-year-old man sought care for dyspnea and chest pain for 72 hours; he had evidence of global cardiac decompensation for a severe ischemic heart disease with preserved left ventricular ejection fraction. Cardiac blood marker analysis revealed an increased troponin level to 250 ng/L and thereafter 350 ng/L (reference range <14 ng/L). Electrocardiogram results showed ST-segment depression in the lateral leads. In this context of non–ST-segment elevation myocardial infarction, the patient was hospitalized in the cardiology unit. On day 6, transthoracic echocardiography revealed an aortic valve bioprosthesis, reshaped, with a thickening of the cusps and a vibratory element attached on the ventricular side (7 × 4 mm), suggesting vegetation suspicious for infective endocarditis (Appendix Figure). The patient became febrile. We collected a total of 7 sets of aerobic and anaerobic blood bottle cultures during days 9–12; all showed a Gram-positive coccus in clusters. Matrix-assisted laser desorption/ionization time-of-flight mass spectrometry identification (VitekMS; bioMérieux, https://www.biomerieux.com) indicated *S. succinus* with a 99.9% index. 

The patient initially received 6 g intravenous cefazolin; on day 13 we changed the antimicrobial treatment to intravenous daptomycin (10 mg/kg) and gentamicin (3 mg/kg) every 48 h. Finally, after a dedicated endocarditis multidisciplinary consultation, we changed the patient’s regimen on day 22 to daptomycin (10 mg/kg) and rifampin (900 mg) for 6 weeks. The patient returned home; follow-up care was scheduled with a hospital at home. The patient outcome was favorable without relapse or side effects from daptomycin/rifampin. His last cardiology appointment was 11 months after his initial treatment; no sequelae of endocarditis were present.

*S. succinus* susceptibility testing was a challenge. We performed methicillin resistance testing with cefoxitin screen and oxacillin testing using the AST-P668 bioMérieux card with a VitekXL automated system. However, we observed a discrepancy between the results from the 2 tests. To confirm oxacillin resistance, we tested by agar diffusion method using impregnated disks and interpreted them in accordance with EUCAST (European Committee on Antimicrobial Susceptibility Testing) criteria (https://www.eucast.org/fileadmin/src/media/PDFs/EUCAST_files/Breakpoint_tables/v_13.0_Breakpoint_Tables.pdf). We used oxacillin (1 μg) and cefoxitin (30 μg) disks (Bio-Rad, https://www.bio-rad.com). The oxacillin (1 μg) disk diffusion method detected oxacillin resistance. In contrast, the isolate was susceptible when we used the cefoxitin (30 μg) disk test. In addition, we performed an oxacillin MIC strip test; MIC of 0.5 (mg/L), indicated that the strain was susceptible according to the EUCAST 2022 criteria.

A retrospective study (*5*) of penicillin-binding protein (PBP) assays indicating antimicrobial drug resistance has shown that preinduction with cefoxitin/oxacillin and reading of the test after 10 min (instead of 5 min) substantially improve the sensitivity, specificity, and robustness of the immunochromatographic assay PBP2a (Abbott, https://www.globalpointofcare.abbott) for coagulase-negative staphylococci. We performed PBP2a detection from bacterial culture after a preinduction with cefoxitin, but results were negative. Thereafter, we performed *mecA* gene detection by PCR to identify oxacillin-resistant *Staphylococcus* (*6*); however, we did not detect the *mecA* gene by PCR.

Finally, we sent the isolate to the French Reference Center for *Staphylococci* (Lyon, France) on day 19 for detection of other *mec* genes; this test result was negative. Staff at the reference center performed whole-genome sequencing of the strain as previously described (*7*); results revealed no site-specific insertion sequences comprising direct-repeat sequences typical of a staphylococcal cassette chromosome–like cassette (*8*). To evaluate the possibility of resistance by PBP modification, we performed a disk diffusion method for antimicrobial susceptibility of imipenem (PBP1), cefotaxime (PBP2), oxacillin (PBP3), and cefoxitin (PBP4) (*9*,*10*). The cefotaxime diameter was reduced, indicating resistance in a strain, most likely by a modification of PBP2 (Figure; Appendix Table).

**Figure:**
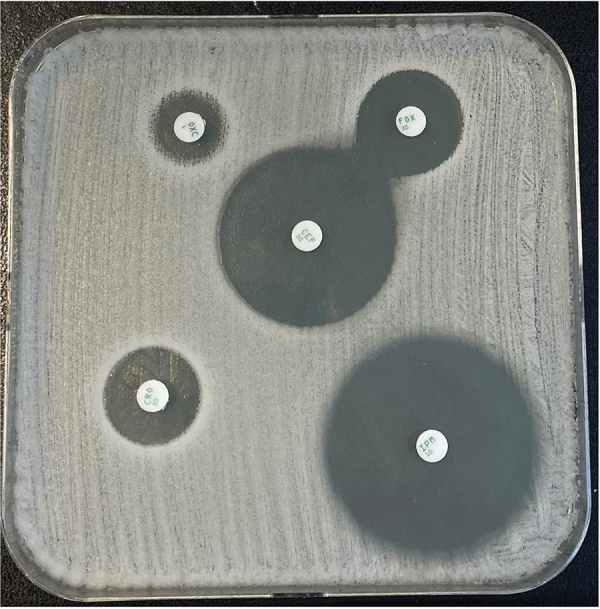
Disk diffusion testing for antimicrobial susceptibility testing of *Staphylococcus succinus* from a patient in France with infective endocarditis. Agar diffusion method using impregnated disks was interpreted according to the criteria of the European Committee on Antimicrobial Susceptibility Testing 2022 (version 13.0) breakpoints for oxacillin susceptibility testing (https://www.eucast.org/fileadmin/src/media/PDFs/EUCAST_files/Breakpoint_tables/v_13.0_Breakpoint_Tables.pdf). CEF, cephalotin; CRO: cefotaxime (PBP2); FOX, cefoxitin (PBP4); IMP, imipenem (PBP1); OXC, oxacillin (PBP3); PBP, penicillin-binding protein.

In conclusion, we identified environmental *S. succinus* behaving as an opportunistic pathogen as the cause of infective endocarditis in a patient with many cardiovascular risk factors. The source of *S. succinus* was not clearly established. Virulence factors contributing to *S. succinus* pathogenicity are not yet well defined. We further described the difficulty of determining the resistance profile of this rarely pathogenic species mimicking either the borderline oxacillin-resistant *S. aureus* phenotype with an elevated oxacillin MIC value, or to a lesser extent the modified *S. aureus* phenotype in the absence of *mec* gene–mediated resistance. Our findings highlight the importance of a multiple-technology approach for laboratories assessing methicillin resistance using a combination of phenotypic and genotypic methods.

Appendix Additional information about a case of *Staphylococcus succinus* infective endocarditis in France.
