# Supplementary material for: Staphylococcus succinus Infective Endocarditis, France
Source: Emerg Infect Dis. 2024 Mar;30(3):601–3. doi: 10.3201/eid3003.230986 (PMC10902547; doi:10.3201/eid3003.230986)
Supplement: Appendix — Additional information about a case of Staphylococcus succinus infective endocarditis in France. [file 23-0986-Techapp-s1.pdf]

Article DOI: http://doi.org/10.3201/eid3002.230986

EID cannot ensure accessibility for supplementary materials supplied by authors. Readers who have difficulty accessing supplementary content should contact the authors for assistance.

Staphylococcus succinus Infective Endocarditis, France

Appendix

Appendix Table. Antibiotic susceptibility testing according to EUCAST V12.0*

Test	Antibiotics	Interpretation (MIC mg/L)	Abbott PLP2a	Nantes in-house PCR *mecA*	Nantes Cepheid *mecA*	Lyon in-house PCR *mecA*	Lyon in-house PCR *mecC*	Lyon WGS analysis
AST-P335 bioMérieux card	Cefoxitin screen	Positive						
	Oxacillin	[R] (0.5)	Negative	Negative	Negative	Negative	Negative	No *mec*, no *SSCmec*
	Kanamycin	S (<4)						
	Gentamicin	S (<0.5)						
	Levofloxacin	I (0.5)						
	Vancomycin	S (2)						
	Erythromycin	R						
	Clindamycin	R						
	Fusidic Acid	S (<0.5)						
	Trimethoprim/ sulfamethoxazole	S (<10)						
	Fosfomycin	R						
	Tetracyclin	S (<1)						
	Linezolid	S (<2)						
	Rifampicin	S (0.03)						
MIC strip test	Ceftobiprole	S (0.5)						
	Ceftaroline	S (0.19)						

*WGS data. The data for this study have been deposited in the European Nucleotide Archive (ENA) at EMBL-EBI under accession number PRJEB66420 (https://www.ebi.ac.uk/ena/browser/view/PRJEB66420). Characterization of the SCC elements was performed by searching for ISSs characteristic of SCC elements and associated direct and inverted repeats.

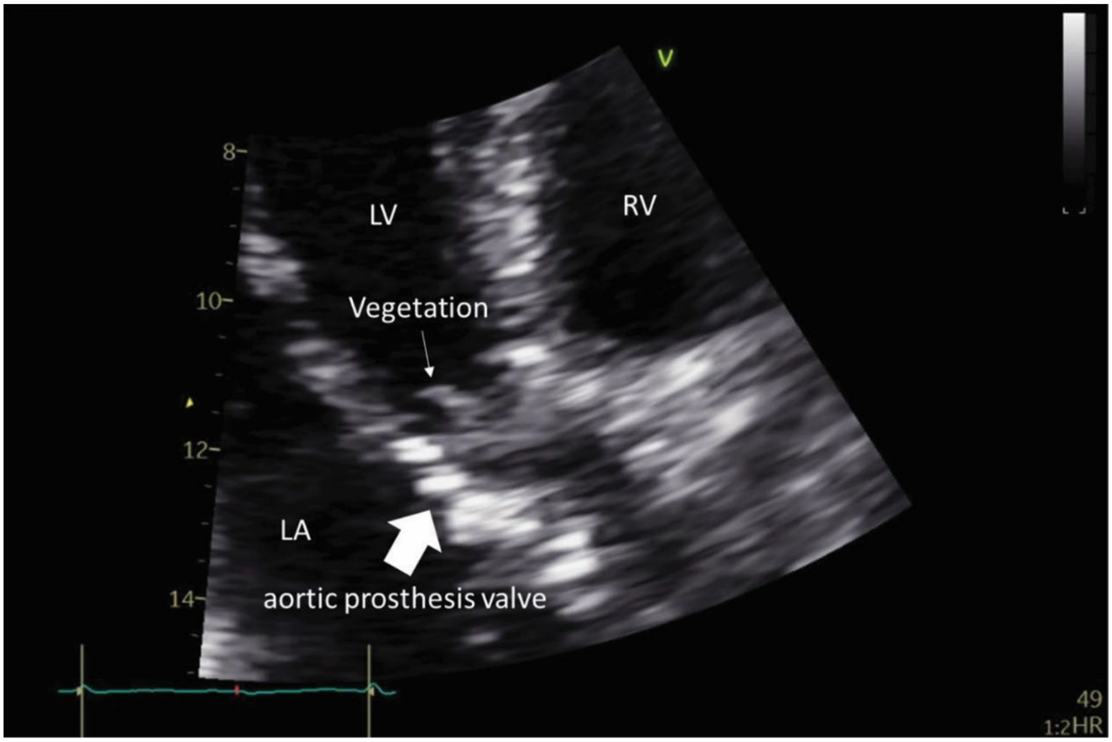

Appendix Figure. Transthoracic echocardiogram. Image shows vegetation on the aortic valve bioprosthesis. LA, left atrial; LV, left ventricle; RV, right ventricle.
